# Key Recommendations for Antimicrobial Resistance Surveillance: Takeaways From the CAPTURA Project

**DOI:** 10.1093/cid/ciad487

**Published:** 2023-12-20

**Authors:** Ahmed Taha Aboushady, Mohammad Julhas Sujan, Kien Pham, Adam Clark, Florian Marks, Marianne Holm, Hea Sun Joh, Nimesh Poudyal, John Stelling

**Affiliations:** Brigham & Women's Hospital, Harvard Medical School, Boston, Massachusetts, USA; International Vaccine Institute, Seoul, Republic of Korea; International Vaccine Institute, Seoul, Republic of Korea; International Vaccine Institute, Seoul, Republic of Korea; Brigham & Women's Hospital, Harvard Medical School, Boston, Massachusetts, USA; International Vaccine Institute, Seoul, Republic of Korea; Cambridge Institute of Therapeutic Immunology and Infectious Disease, University of Cambridge School of Clinical Medicine, Cambridge, United Kingdom; Heidelberg Institute of Global Health, University of Heidelberg, Heidelberg, Germany; Madagascar Institute for Vaccine Research, University of Antananarivo, Antananarivo, Madagascar; International Vaccine Institute, Seoul, Republic of Korea; International Vaccine Institute, Seoul, Republic of Korea; International Vaccine Institute, Seoul, Republic of Korea; Brigham & Women's Hospital, Harvard Medical School, Boston, Massachusetts, USA

**Keywords:** antimicrobial resistance, surveillance, data and laboratory quality management, South Asia, Southeast Asia

## Abstract

Antimicrobial resistance (AMR) is a growing global public health challenge associated with 4.95 million deaths in 2019 and an estimated 10 million deaths per year by 2050 in the absence of coordinated action. A robust AMR surveillance system is therefore required to avert such a scenario. Based on an analysis of country-level AMR data in 8 Capturing Data on Antimicrobial Resistance Patterns and Trends in Use in Regions of Asia (CAPTURA) countries, we present a list of key recommendations to strengthen AMR surveillance. We propose 10 primary considerations under 3 broad categories, including recommendations on (1) laboratory and testing practices, (2) data management and analysis, and (3) data use.

## Teaser

Antimicrobial resistance (AMR) is a rapidly growing global public health challenge. AMR data entry, management, analysis, and use need extensive improvement and quality assurance. Stakeholders including government agencies have a critical role in strengthening sustainable and robust AMR surveillance systems.

## THE NEED FOR AMR SURVEILLANCE SYSTEMS IN SOUTH AND SOUTHEAST ASIA

Antimicrobial resistance (AMR) is a global public health challenge associated with increasing morbidity, mortality, and cost [1, 2]. According to one predictive model, bacterial AMR was associated with 4.95 million deaths in 2019 [3] and is expected to cause >10 million deaths per year by 2050 in the absence of stringent action [4]. Low- and middle-income countries (LMICs) are disproportionately impacted with the highest AMR burden [5, 6]. This is associated with high prevalence of infectious diseases, scarce human and technical resources, and fragile health policies [7], resulting in antibiotic overuse and misuse. Although this is primarily due to the nonprescription use of antimicrobials [8], prescriptions also have a role due to the lack of appropriate diagnostics, well-informed guidelines, and financial incentives [9]. The World Health Organization (WHO) Southeast Asia region is classified to be at highest risk of AMR, and governments in these countries are proactively working at the policy end. Actions already taken include adopting the Jaipur Declaration on Antimicrobial Resistance in 2011 and making AMR a flagship priority in 2014 [10].

AMR surveillance is a cornerstone in the AMR response as it defines the scope of the problem of drug resistance and identifies interventions to enhance the appropriate use of antimicrobials [11]. In recent years, significant technological developments have been made in the field of AMR to inform policies and support laboratory-based activities, including the use of genomic technologies to track AMR [12]. However, these developments have yet to reach most LMICs [13, 14], where there is an urgent need to establish robust AMR surveillance systems to support procedures to detect early warning, tailor responses, and analyze epidemiological trends [15]. Electronic healthcare data management tools must be reliable and adaptable to provide seamless and automated functionality to support AMR surveillance systems [16]. While such methods should ensure data privacy and provide more validated processes to control data quality and completeness (targeting zero-defect automated reporting) [17], the collected information should strengthen laboratory operations and decisions. Evidence-based decision-making reinforces that decisions should be based on the best available records from research that is key to the design of AMR control and prevention activities. In addition, the decision-making process should consider factors such as context; public opinion; safety; effectiveness; impact on equity; acceptability to stakeholders; and the feasibility, affordability, and sustainability of proceedings. This improves transparency and accountability and impacts activities to better understand the AMR situation and inform health policies [18].

The Capturing Data on Antimicrobial Resistance Patterns and Trends in Use in Regions of Asia (CAPTURA) project was tasked with expanding the volume of data in Asia by collecting retrospective antimicrobial resistance, use, and consumption data and, while doing so, evaluating data quality and facility capacity. Evaluating AMR data (eg, antimicrobial susceptibility testing [AST] records) and the use of data in decision-making (eg, clinical practices, data management, guidelines) were integral to the project in South Asian and Southeast Asian countries. The program is funded by the Fleming Fund and is led by the International Vaccine Institute in collaboration with the Brigham and Women's Hospital, the Big Data Institute of the University of Oxford, and the Public Health Surveillance Group [19].

We analyzed primary data on AMR using the WHONET (www.whonet.org) system, an open-access software used to curate information on AST results [20]. Based on our activities, including retrospective data analysis on AMR and rigorous consultation with stakeholders and experts in participating countries, we propose 10 primary considerations under 3 broad categories, including recommendations on (1) laboratory and testing practices, (2) data management and analysis, and (3) data use for effective AMR surveillance [21–24]. The WHONET system addresses many of these challenges, with others requiring extensive intervention in current laboratory, data entry, and use practices. We are confident these recommendations enhance and support the local and national surveillance of AMR to generate quality data for interpretation, policy adoption, and intervention.

## RECOMMENDATIONS TO STRENGTHEN AMR SURVEILLANCE SYSTEMS

**Figure 1.**
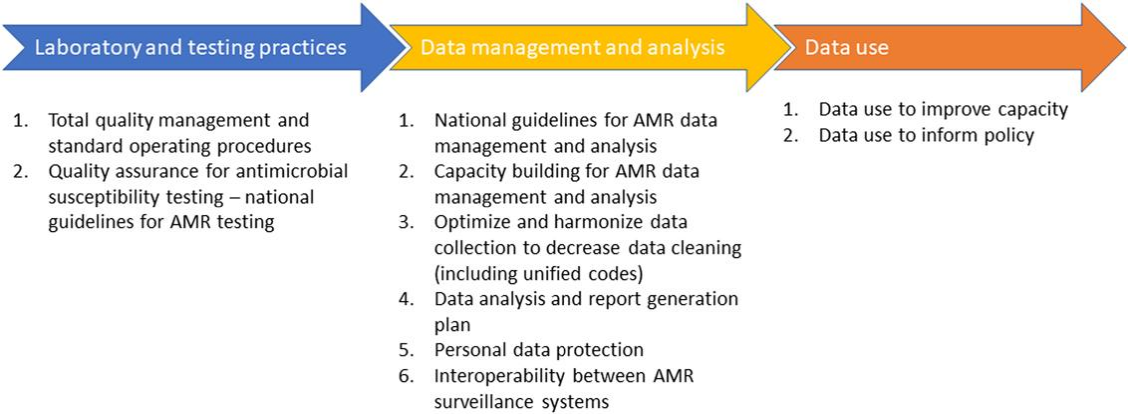
Summary of the key recommendations from the Capturing Data on Antimicrobial Resistance Patterns and Trends in Use in Regions of Asia (CAPTURA) project. Abbreviation: AMR, antimicrobial resistance.

We make 3 key recommendations based on the 3 main themes identified (Figure 1).

### Recommendations on Laboratory and Testing Practices

#### Total Quality Management and Standard Operating Procedures

**Figure 2.**
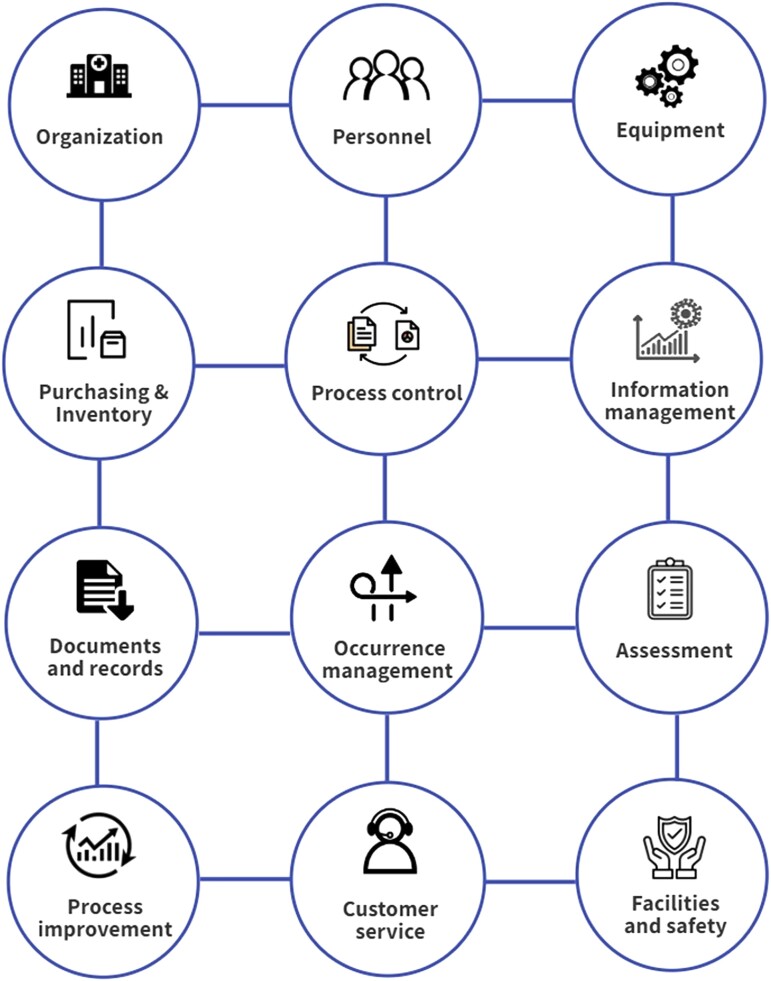
The list for laboratory quality essentials. Source: World Health Organization, 2016 [26].

During our review of laboratory practices and capacities, many countries lacked clear laboratory quality management systems and standard operating procedures (SOPs). These features are necessary to monitor the reliability of all aspects of laboratory operations. The primary goal of such quality management systems is the continuous improvement of laboratory processes, which must be done systematically [25]. A quality management system produces improved laboratory practices that efficiently detect errors and prevent them from recurring. The lack of such a system leads to poor-quality management results with unnecessary treatment, incorrect and suboptimal therapy decisions, delayed diagnosis, and unnecessary follow-up diagnostic testing, all of which have been observed in some countries [26]. Errors and problems may go undetected without a laboratory quality management system. The system needs to be comprehensive and cover all 12 laboratory quality essentials as described by WHO [26] (Figure 2).

Furthermore, we recommend that the laboratory quality management system be accompanied by SOPs to provide clear instructions on routine laboratory processes and keep them updated based on performance monitoring, facilitated by the system [27]. During team visits, we noticed that some laboratories had SOPs, which personnel were not adequately trained to implement. Thus, training new personnel and refreshing the skills and knowledge of established personnel is crucial for the successful adoption and implementation of quality management systems and SOPs.

Last, laboratories should routinely test relevant quality control strains from the American Type Culture Collection (ATCC) or other reference systems, including but not limited to ATCC 25922 *Escherichia coli* and ATCC 25923 *Staphylococcus aureus* [28]. In addition, laboratories should identify and systematically test relevant pathogen-specific antimicrobial panels following guidance from reference method authorities. Such referenced control testing results should be recorded safely to allow for periodic evaluation function in the laboratory processes.

#### Quality Assurance for AST: National Guidelines for AMR Testing

While we provided assistance to different countries, many developed national AMR testing standards in an effort to improve the frequency of laboratory testing; however, some countries still lack such guidelines. These policies must provide clear instructions on priority testing for common species and consider different antimicrobial classifications published by WHO and other international organizations. Guidelines should also be based on internationally accredited standards, such as the Clinical and Laboratory Standards Institute and the European Committee on Antimicrobial Susceptibility Testing, and be adapted to local contexts and known AMR patterns and trends. Policies must be flexible in dynamic situations in which information needs, resources, and technology evolve and should be periodically revisited and reevaluated to ensure they continue to meet evolving circumstances. Guidelines must include all health sectors, including humans, animals, food, and the environment. Such inclusivity would ensure a well-integrated and connected surveillance system that can prevent, track, and respond to antimicrobial susceptibility in a coordinated manner.

Moreover, knowledge is not the only challenge that hinders the regularity of AST. Many countries report frequent stock shortages of panels and other required reagents. The lack of reliable sources of quality materials influences the quality, reliability, and generalizability of data and trend analysis. In addition, different issues in the supply chain often make panels unavailable. Accordingly, we hope that the exact problems can be identified and addressed by implementing laboratory quality management systems. In many countries, this goes beyond laboratories themselves, and support from policymakers is essential to prioritize the AMR surveillance agenda. Further to availability, the standards of proposed supplies need to be specified to ensure that only nonexpired media and materials meeting quality standards are used for testing [29].

To address the beforementioned quality issues in South and Southeast Asian laboratories, another Fleming Fund–funded project, EQAsia, was developed to strengthen the provision of external quality assessment services across the One Health sector in South and Southeast Asia [30].

### Data Management and Analysis Recommendations

#### National Guidelines for AMR Data Management and Analysis

AMR data management and analysis were troublesome in many of the datasets reviewed by the team. For example, data triangulation from multiple laboratories was complicated and often required manual data reviews and cleaning. This difficulty was due to inconsistent data fields, distinct codes such as antibiotics, organisms, specimens, incomplete entries, use of free text, and so on. Accordingly, AMR coordination committees, or equivalent multisectoral public bodies, should develop national guidelines for AMR data management and analysis to ensure that data are better managed and, thus, more consistent, facilitating analysis and interpretation [31]. Additionally, this should include the minimum data required for AMR surveillance management [32] and highlight the roles of the multidisciplinary personnel involved in the process.

#### Capacity Building for AMR Data Management and Analysis

The team noticed a gap in knowledge and capacity in some countries for AMR data management and analysis. Accordingly, national guidelines for AMR data management and analysis should be complemented by capacity building of laboratory teams to ensure they are equipped with the necessary knowledge and skills to fulfill the objectives of the AMR surveillance system. This should include training and experience in data entry, management, analysis, interpretation, outbreak investigation, and response. Moreover, a national master trainer pool should be developed to ensure smooth laboratory support and to improve the sustainability of skills. This can be achieved through the training and retention of expert trainers and technical resource personnel who can lead further team training.

#### Optimize and Harmonize Data Collection to Decrease Data Cleaning Requirements

Standardization of data values and formats has posed a significant challenge to the project team, requiring detailed reviews of hundreds of thousands of records and meticulous cleaning to ensure that most entries are captured completely, accurately, and consistently. The data cleaning stage can be tedious but is essential. For better facilitation and optimization, we recommend using standardized data entry and management approaches and ensuring the completeness of entries and results. Many countries have complicated and fragmented data ecosystems, with laboratories in one nation using different laboratory information systems and assorted data structures with varying extents of free text, thus preventing interlaboratory surveillance and national data collection. Therefore, we recommend investing in nationwide data collection and input standards to facilitate the achievement of national AMR surveillance objectives.

To achieve the aspired data quality, a data validation process is necessary to harmonize terminology and routines and to promote collaboration between laboratories in the same network in order to correct inconsistencies and errors [33]. For the CAPTURA project, a publicly available WHONET code search engine within the BacLink import tool facilitates mapping relevant codes for organisms, antibiotics, and specimens, utilizing WHONET code lists [34].

#### Data Analysis and Report Generation Plan

We recommend having a clear way forward for data analysis and utilization. Thus, data should be collected with an analysis plan and reporting framework in mind. The data collected should be based on the reporting plan to ensure that all recommended fields are captured during the data entry stage, facilitating subsequent steps to utilize the data. Meanwhile, laboratories that already collect routine data should reevaluate their current data collection approach to ensure that it meets their needs and purposes.

The scope of the CAPTURA project involved the collection, analysis, and interpretation of historical data. The retrospective nature of CAPTURA data and recommendations underline the importance of timely and prompt use of the data to recognize and confirm emerging threats, enhance clinical outcomes, and develop updated surveillance and treatment guidance [35].

#### Personal Data Protection

AMR laboratory data include personal identifiers and details that must be protected and appropriately managed. We have observed that many datasets are circulated with more personal identifiers, such as patient names and dates of birth, than are required for analysis purposes. Therefore, laboratory data managers should be trained in secure file management, including the encryption of computers and other storage devices, data encryption, and organizational and governmental requirements. This is necessary for the protection of patient confidentiality and the sharing of protected health information with external organizations, while maintaining data structure and degranulation. In this project, we utilized the WHONET data encryption function, which uses unidirectional encryption, thus preventing any subsequent “decryption” of results [36]. This is of utmost importance when sharing data beyond the facility level, for example, at the national level or with outside organizations.

In addition to file encryption, secure data storage is paramount, limiting data access to only the minimum required personnel. Furthermore, encryption and data sharing should be preceded by a data use/share agreement to ensure appropriate use of the data. Finally, more stringent rules and regulations must be implemented to secure such sensitive data and to prevent similar instances where data are shared without the encryption of personal identifiers.

#### Interoperability Between AMR Surveillance Systems

In many of the countries, we noticed fragmented systems, with several software and duplicating functionalities, that overwhelm surveillance personnel and delay data utilization and reporting. Interoperability is crucial for health data and is a necessary functionality in any surveillance software [37]. We strongly recommend exploring all possible data interoperability options and better understanding the digital ecosystem before introducing any new solution. We understand the challenges imposed by limited resources and thus, such investigation would allow for the optimal use and allocation of available resources.

Interoperability is needed between software used within the same sector and between different sectors. Within human health, interoperability would facilitate the data entry process, prevent duplication of efforts through double data entry in other software, and offer a more automated solution to enable data migration from one software to another. Across sectors, this yields a broader understanding of AMR trends and ensures the reporting of more representative data to international platforms, such as the WHO Global Antimicrobial Resistance and Use Surveillance System (GLASS) [38]. BacLink, an accessory software downloaded with WHONET, can import data from diverse systems to WHONET-compatible datasets that can be combined, encrypted, and analyzed with other datasets [39].

### Data Use Recommendations

#### Data Use to Improve Capacity

We have noticed a huge gap in data use. Many countries curate large amounts of data but with only limited scope for analysis reports. Data should be used to inform capacity development and national health planning. For internal improvement, we recommend that more frequent and regular reports are necessary to monitor and improve laboratories. We recommend that reporting be at least monthly at the facility level and quarterly at the national level. These reports can be used by laboratory managers and/or central level management as part of their routine monitoring activities to ensure the prompt detection of deficiencies in laboratory practices, quality of testing materials, data management, and alarming trends that require immediate action. Additionally, it is essential to identify incorrect testing practices that need to be addressed, such as reporting nitrofurantoin for bloodstream isolates [40] or unlikely test results suggestive of deficiencies in test performance or reagent quality (eg, *Escherichia coli* susceptible to ampicillin but resistant to amoxicillin/clavulanic acid). Inaccurate testing drains resources and increases patient wait time and can cause physical or psychological harm, decrease confidence in laboratory staff, and lead to ineffective treatment [41].

Moreover, while these reviews may identify laboratory or data quality issues, as mentioned above, laboratory quality management system and data validation processes need to be in place and periodically reviewed to evaluate performance and identify solutions to challenges. Furthermore, the findings should be used to create automated quality control alerts in the laboratory information system. The WHONET automatic alert function, which was used to test the usefulness of such an approach on retrospective analysis, would introduce prompt notification and action by laboratory and infection control if implemented in real time. Finally, at the national level, such reviews and data would help guide resource allocation to maximize efficiency through understanding laboratory capabilities, resources, and needs.

#### Data Use to Inform Policy

Similarly, we recommend that data go beyond the interpretation level, to be translated and used at a higher level to inform policymaking. Evidence-informed decision-making is a cornerstone for efficient and inclusive health systems [42]. Therefore, microbiology data need to be translated into clear, understandable language for decision-makers to enable utilization of the data. The process needs to start by identifying the appropriate One Health stakeholders involved, identifying the data needs of distinct sectors and audiences, and developing a communication strategy to share the findings. Including representatives from all sectors is crucial to ensure equal participation. Furthermore, data systems should be agile and tailored to important resistance and epidemiological findings. For example, with significant outbreaks of *Salmonella* Typhi in Nepal, national stakeholders requested the preparation of specific reports that would provide valuable overviews and insights that can be used to support outbreak response and control.

Furthermore, we hope the data can go beyond the national level and support regional AMR networks. This should strengthen regional preparedness and response, increasing alertness when any outbreak happens in the area. This would align with the core principles of the International Health Regulations (2005) [43].

## CONCLUSIONS

In conclusion, with a national and local focus, we have highlighted recommendations to strengthen AMR surveillance programs by optimizing data entry, management, analysis, interpretation, and use. We have proposed 10 main recommendations divided into 3 themes. First, laboratory and testing practice recommendations to implement a laboratory quality management system and develop national guidelines for AMR testing based on national standards, quality control standards, and available resources. Second, data management and analysis recommendations to build capacities and develop AMR data management and analysis guidelines, address data entry issues, including code harmonization, interoperability and personal data protection, and create an AMR data analysis and reporting plan. Finally, data use recommendations to improve laboratory capacity through periodic reviews, addressing incorrect testing practices and national health planning, providing evidence for policymaking, One Health integration, and supporting regional data sharing and collaboration.

The above recommendations provide essential considerations for strengthening AMR surveillance programs, acknowledging that surveillance systems need to be tailored and context specific. This cannot be a “one size fits all” solution. All of the above need to be coupled with enhanced communication and strategic management at local, national, and regional levels to facilitate feedback and improve procedures and local user acceptability.

## Notes

***Disclaimer.*** The views expressed here are those of the authors and not necessarily those of the UK Department of Health and Social Care or its management agent, Mott MacDonald; and, International Vaccine Institute.

***Supplement sponsorship.*** This article appears as part of the supplement “Capturing Data on Antimicrobial Resistance Patterns and Trends in Use in Regions of Asia (CAPTURA),” funded by the Department of Health and Social Care's Fleming Fund using UK aid.

***Financial support.*** This work was supported by the Fleming Fund regional grants (grant numbers FF10-135 and FF11-139). The Fleming Fund is a UK aid investment to support surveillance of antimicrobial resistance in low- and middle-income countries in Africa and Asia. The regional grants program is managed by Mott MacDonald Ltd, the Fleming Fund Grants Management Agent.
